# Dose of antivenom for the treatment of snakebite with neurotoxic envenoming: Evidence from a randomised controlled trial in Nepal

**DOI:** 10.1371/journal.pntd.0005612

**Published:** 2017-05-16

**Authors:** Emilie Alirol, Sanjib Kumar Sharma, Anup Ghimire, Antoine Poncet, Christophe Combescure, Chabilal Thapa, Vijaya Prasad Paudel, Kalidas Adhikary, Walter Robert Taylor, David Warrell, Ulrich Kuch, François Chappuis

**Affiliations:** 1 Division of Tropical and Humanitarian Medicine, University Hospitals of Geneva, Geneva, Switzerland; 2 B.P. Koirala Institute of Health Sciences, Dharan, Nepal; 3 Clinical Research Centre, University Hospitals of Geneva, Geneva, Switzerland; 4 Dumkauli Primary Health Care Centre, Nawalparasi, Nepal; 5 Bharatpur Hospital, Chitwan, Nepal; 6 Mahidol Oxford Research Unit, Bangkok, Thailand; 7 Nuffield Department of Clinical Medicine, University of Oxford, Oxford, United Kingdom; 8 Institute of Occupational Medicine, Social Medicine and Environmental Medicine, Goethe University, Frankfurt am Main, Germany; Liverpool School of Tropical Medicine, UNITED KINGDOM

## Abstract

**Background:**

Currently, there is inadequate evidence on which to base clinical management of neurotoxic snakebite envenoming, especially in the choice of initial antivenom dosage. This randomised controlled trial compared the effectiveness and safety of high versus low initial antivenom dosage in victims of neurotoxic envenoming.

**Methodology/ Principal findings:**

This was a balanced, randomised, double-blind trial that was conducted in three health care centers located in the Terai plains of Nepal. Participants received either low (two vials) or high (10 vials) initial dosage of Indian polyvalent antivenom. The primary composite outcome consisted of death, the need for assisted ventilation and worsening/recurrence of neurotoxicity. Hourly evaluations followed antivenom treatment. Between April 2011 and October 2012, 157 snakebite victims were enrolled, of which 154 were analysed (76 in the low and 78 in the high initial dose group). Sixty-seven (43·5%) participants met the primary outcome definition. The proportions were similar in the low (37 or 48.7%) vs. high (30 or 38.5%) initial dose group (difference = 10·2%, 95%CI [-6·7 to 27·1], p = 0·264). The mean number of vials used was similar between treatment groups. Overall, patients bitten by kraits did worse than those bitten by cobras. The occurrence of treatment-related adverse events did not differ among treatment groups. A total of 19 serious adverse events occurred, including seven attributed to antivenom.

**Conclusions:**

This first robust trial investigating antivenom dosage for neurotoxic snakebite envenoming shows that the antivenom currently used in Nepal performs poorly. Although the high initial dose regimen is not more effective than the low initial dose, it offers the practical advantage of being a single dose, while not incurring higher consumption or enhanced risk of adverse reaction. The development of new and more effective antivenoms that better target the species responsible for bites in the region will help improve future patients’ outcomes.

**Trial registration:**

The study was registered on clinicaltrials.gov (NCT01284855) (GJ 5/1)

## Author summary

Snakebite is an important medical problem in tropical regions, including in Nepal where tens of thousands of people are bitten every year. Snakebite can result in life-threatening envenoming and correct identification of the biting species is crucial for doctors to choose appropriate treatment and anticipate complications. This paper compares two different doses of antivenom for the treatment of neurotoxic snakebite envenoming. Out of 157 snakebite victims presenting to one of the study centers, 78 received a low initial dose and 79 received a high initial dose. The proportion of patients who either died, needed breathing support or additional doses of antivenom were the same in the two groups. Overall, patients bitten by kraits did worse than those bitten by cobras. The occurrence of adverse reactions was comparable among those received low or high initial dose respectively. This study is the first to use a rigorous and robust method for comparing doses of antivenom in snakebite victims in South Asia. Although the high initial dose was not more effective than the low initial dose, it offers the practical advantage of being simpler to administer and was as safe as the low initial dose. The development of new and more effective antivenoms that better target the species responsible for bites in the region will help improve future patients’ outcomes.

## Introduction

Snakebite envenoming is a neglected disease *par excellence* that primarily affects poor communities in the tropics, but attracts little interest from pharmaceutical companies, health agencies and research funding bodies. This neglect has resulted in a paucity of scientific evidence on which to base therapeutic decisions and support robust guidelines. The antivenom development pipeline remains desperately dry [1,2]. Moreover, the absence of rigorous regulatory oversight has resulted in the marketing of antivenom of doubtful efficacy and variable quality and safety [1–3]. The optimal dosage of antivenom is highly debated. Antivenom potency varies widely, and the initial doses recommended by manufacturers range from 1 to over 30 vials [4]. Recommendations are usually based on median lethal (LD_50_) and effective dose (ED_50_) assays in which venom and antivenom are incubated *in vitro* before being injected into mice. However, rodent models are poor substitutes for clinical trials [5], and few randomised, dose ranging controlled trials (RCT) of snakebite envenoming have been conducted [6–16]. Moreover, a recent systematic review found that trials conducted in South Asia generated very low quality evidence [17].

In Nepal, snakebite is an important public health problem [18–20] with peak annual incidence and mortality rates of up to 1162/100 000 and 162/100 000, respectively, reported in eastern regions [19]. Snakebite is a disease of poverty. Farmers, plantation workers and herders are the main victims [21]. Elapid snakes, notably the Indian spectacled cobra (*Naja naja*) and the common krait (*Bungarus caeruleus*), cause most cases of snakebite envenoming in Nepal [22,23]. Elapid envenoming is characterized by a progressive, descending neuromuscular paralysis, leading to respiratory failure and death [24,25]. Since 1998, Indian polyvalent antivenom has been provided free of charge to all hospitals in Nepal by the Ministry of Health (MoH). The treatment of envenoming varies widely, with antivenom total doses ranging from 2 to 115 vials [18]. Case fatality rates (CFR) also vary widely, from 3% to 58% [18].

Indian polyvalent antivenom costs between 6·50 to 11·00 US$ per vial [26]. In order to minimise expense, the Nepalese MoH recommends a low initial dose (2 vials) of antivenom as an intravenous (IV) push, followed by an infusion of additional vials titrated to clinical response [27], consistent with some manufacturer guidelines. However, this dosing regimen contrasts with expert opinion which recommends a high loading dose of 10 vials (100 ml) administered as an IV push, arguing that this should neutralize neurotoxins more effectively before they become irreversibly bound to tissue receptors [28,29]. World Health Organization (WHO) guidelines also recommend an initial dose of 10 vials for envenoming after bites by South Asian cobras and kraits [30]. There are no published RCTs addressing the optimal dose of antivenom in neurotoxic envenoming and observational studies are unhelpful [31,32]. Given the lack of data and the need to optimise treatment of snakebite envenoming in Nepal, we conducted an RCT comparing high versus low initial antivenom dose in patients with neurotoxic envenoming.

## Methods

### Ethics statement

Ethical approvals were obtained from the B.P. Koirala Institute of Health Sciences Ethics Committee, the Nepal Health Research Council (NHRC) and the Geneva University Hospitals Ethics Committee. The study was registered on clinicaltrials.gov (NCT01284855). Written informed consent was obtained from all adult participants prior to inclusion, and from guardians for minor participants. Whenever possible assent was also sought from children. For participants who were unable to read and/or write, an independent witness was present during the consent process and signed the consent form next to the participant’s thumbprint.

### Study design

This was a balanced, randomised, double-blind, parallel trial comparing two dosing regimens of antivenom. The study was conducted between April 2011 and March 2013 at the Snake Bite Treatment Centre of Damak Red Cross Society, the Snake Bite Management Centre of Charali, both in Jhapa district, and the Bharatpur District Hospital, Bharatpur, in Chitwan district. All centres are located in the Terai plains of Nepal.

### Participants

Snakebite victims were enrolled in the study if they gave written informed consent (assent if aged 12 to 18) and had ≥1 sign(s) of neurotoxic envenoming: bilateral ptosis; inability to frown, open the mouth, protrude the tongue, or clear secretions; broken neck sign; skeletal muscle weakness (power < 3 UK MRC scale); gag reflex loss; and paradoxical breathing.

Those presenting >24 hours post-bite, with a proven viper bite, who had already received antivenom, or were children below 5 years, pregnant or breast feeding women, individuals with a history of neuromuscular disease, known allergy to horse protein, and those with an immediate need for mechanical ventilation were excluded.

### Randomisation and masking

Randomisation was stratified by centre, and within each stratum, patients were randomized in blocks of variable sizes, according to a computer generated list. Sealed sequentially numbered envelopes containing the antivenom regimen were prepared accordingly. For quality control, 10% of the envelopes were double-checked by an independent statistician.

The envelopes were kept in the site pharmacy, to which only the trial pharmacist had access. Upon inclusion of a trial participant, the pharmacist opened the envelope in sequence. Reconstitution of antivenom, dilution and preparation of push injections and perfusions took place in the pharmacy. To maintain blinding, the total volume and appearance of push injections and infusions were identical in the two study arms. The study clinicians and patients were unaware of treatment allocation. If neurotoxicity persisted or worsened, the clinician asked the pharmacist to prepare additional doses of antivenom, according to the indications found in the randomisation envelope for that patient. This was done in the same concealed manner. Compliance with randomisation and masking procedures was assessed as part of the GCP monitoring of the trial.

### Intervention and trial procedures

We used two batches of lyophilised polyvalent antivenom raised against Indian *Daboia russelii*, *Echis carinatus*, *Bungarus caeruleus* and *Naja naja* venoms, manufactured by VINS Bioproducts Ltd, Hyderabad, India. The neutralizing potency of the antivenom (mg of Indian snake venom neutralized per mL of antivenom) as stated by the manufacturer in the Certificate of Analysis (CoA) was: 0.681 mg and 0.636 mg for *N*. *naja*, 0.541 mg for *B*. *caeruleus*, 0.704 mg for *D*. *russelii*, and 0.612 mg and 0.616 mg for *E*. *carinatus*. Trial participants received either the dose regimen recommended by the Nepalese national protocol (low initial dose group) or a high initial dose as recommended by WHO guidelines (high initial dose group). The low initial dose regimen consisted of an initial dose of 2 vials given by IV push, followed by the infusion of 4 vials over 4 hours. If signs of envenoming persisted after the initial 4 hours, the 4 vial infusion was repeated up to three times. If signs of envenoming persisted after 12 hours, an infusion of 2 vials of antivenom was given over 4 hours, every 4 hours, until recovery. In the case of neurotoxic deterioration, 2 vials were administered by IV push as recommended by national guidelines. To maintain the blinding, the high initial dose regimen was adapted to the administration method used in the low initial dose arm. It consisted of an initial dose of 2 vials given by IV push followed by an 8 vial infusion over one hour and an infusion of saline over 3 hours. If signs of envenoming persisted after these first 4 hours, the saline infusion was repeated, to mimic the infusion given in the low initial dose arm. In the case of deteriorating neurotoxic signs, 5 vials of antivenom were given by IV push. The two regimens are described in S2 Fig. The total number of vials of antivenom administered was restricted to 30, irrespective of treatment allocation.

Patients were hospitalised throughout the treatment period. After initial dosing, clinical evaluation was performed every hour until full recovery. Anaphylaxis was managed by stopping the antivenom immediately and administering intramuscular (IM) adrenaline, IV hydrocortisone and IV chlorphenamine. Oxygen, salbutamol inhalations, or the rapid administration of normal saline were given as indicated clinically. Following the publication of an RCT showing the benefits of subcutaneous adrenaline premedication [33], we adopted this strategy after April 2012.

Three follow-up visits were scheduled to assess short and medium-term patient outcome: 7 days, 21 days and 6 months after hospital discharge.

### Outcomes

The primary effectiveness endpoint was a combination of (a) in-hospital death, (b) the need for assisted ventilation and (c) worsening or recurrence of neurotoxicity after the initial dose of antivenom.

The clinical indications for intubation and assisted ventilation were (1) absent gag reflex, (2) presence of paradoxical breathing, (3) respiratory distress or cyanosis, whichever was detected first, and/or (4) oxygen saturation <90% despite high flow oxygen supplementation.

The primary composite endpoint was evaluated at each clinical evaluation, i.e., every hour until full recovery from neurotoxic envenoming. If a patient presented at least one of the sub-criteria, the primary endpoint was deemed positive. If all the sub-criteria were indicated as being absent until full recovery, the primary composite endpoint was deemed negative. Patients with missing data in one of the sub-criteria always presented with at least one other sub-criterion, enabling us to define presence of the primary composite outcome for all patients.

Secondary endpoints included time to recovery and number of antivenom vials used. The safety endpoints were incidence of adverse events (AEs) and serious adverse events (SAEs).

The evolution of neurotoxicity was assessed by a scoring method (S1 Fig). Worsening of neurotoxicity was defined as (1) appearance of ≥ 2 new signs, or (2) appearance of one severe sign (i.e., loss of gag reflex or paradoxical breathing). Persistence of neurotoxicity was defined as the persistence of ≥ 1 sign/s in the absence of criteria of neurotoxicity worsening. Patients were assessed hourly until signs of neurotoxicity disappeared (i.e., clinical score = 0). Complete neurological recovery was defined as reaching and sustaining a score of 0.

Dead snakes brought by victims were labelled and preserved in ethanol. Blinded identification was conducted by taxonomic experts. Morphological features of snakes and mitochondrial cytochrome b sequences of snakes generated using trace DNA swabbed from bite sites were analysed by comparison with reference specimens in museum collections and existing nucleotide sequence databases [23].

### Sample size, and statistical analyses

We assumed a 60% rate of the composite primary outcome in the low dose group, and hypothesised that this would be reduced to 40% in the high dose group. Therefore, 99 patients would be needed in each arm (1-β = 80%, two-sided α = 0·05) and, assuming a dropout rate of 20%, the total estimated sample size was 250 patients.

Effectiveness and safety analyses were performed on a modified intention-to-treat (mITT) population, i.e., all patients who received antivenom and had at least one post-baseline effectiveness evaluation. Analyses were also performed on a per-protocol (PP) population to support conclusions made using the mITT population. The PP population was defined by comparing the total dose of antivenom that participants should have received based on the treatment allocation and their clinical evolution, to the actual total dose administered.

We described patients’ baseline characteristics overall and per treatment arm as frequencies for categorical data and median and inter-quartile ranges (IQR) or means and standard deviations (SD), as appropriate, for continuous data.

Categorical data were compared using Chi-squared or Fisher’s exact test, as appropriate. Continuous data were compared using the Student t or the Mann Whitney U tests.

Survival analyses were conducted (1) on the time free from primary endpoint; (2) on the time to reach a neurotoxicity score of 0. For the latter, fatal cases were included and considered as having never recovered. Survival estimates were obtained with the Kaplan-Meier estimator and the comparison between groups was performed using a log-rank test stratified by center. Adverse Events (AEs) were compared between treatment arms using Fisher’s exact test. All AEs were coded using the MedDRA dictionary version 17·0.

Except for the safety endpoints (AEs and SAEs) for which missing values were considered as no events, no missing data imputation was used.

A two-sided p value ≤ 0·05 was considered statistically significant for all analyses. All analyses were performed on R software (R foundation for Statistical Computing, Vienna, Austria, URL http://www.Rproject.org).

## Results

**Fig 1:**
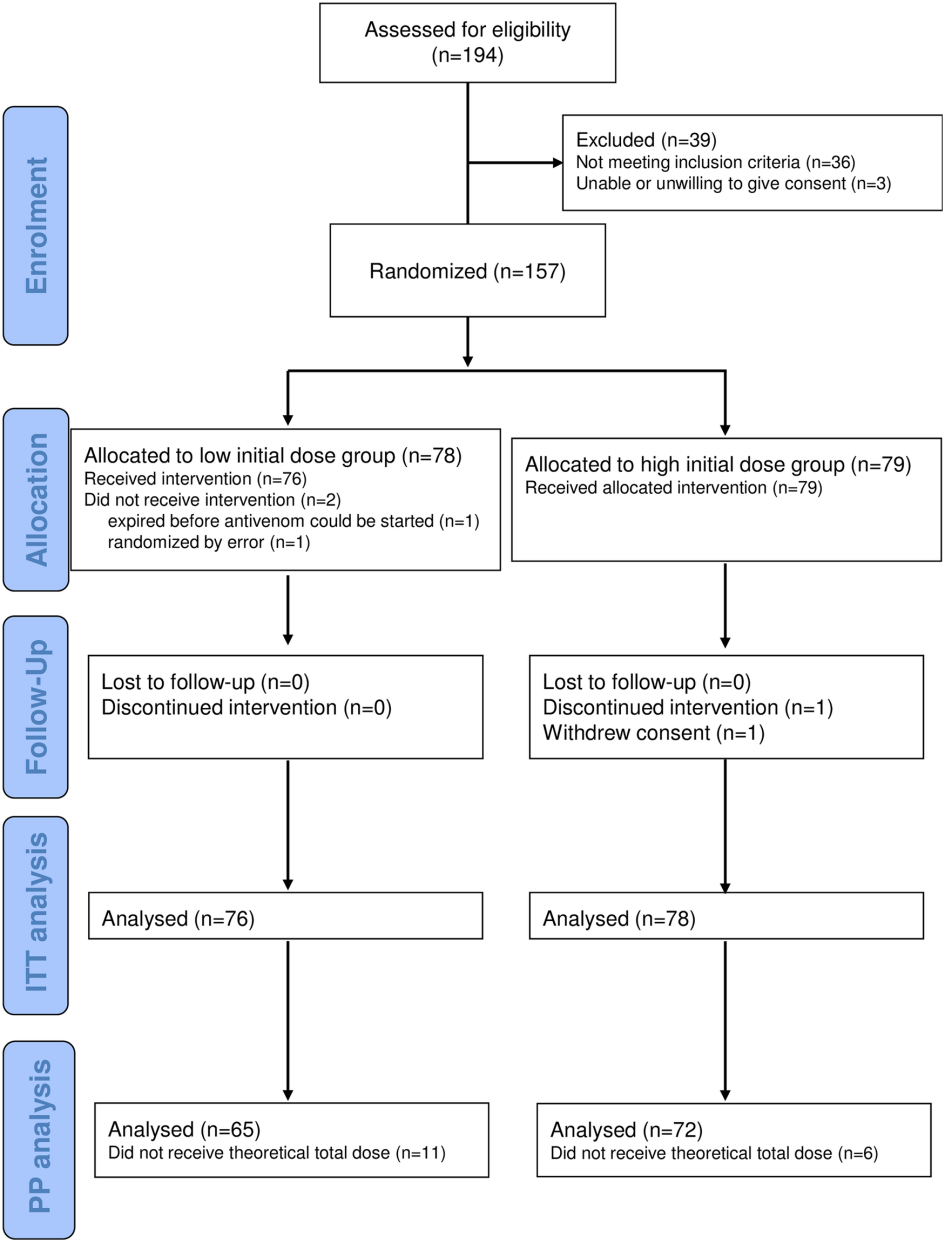
Flow diagram of the progress of participants through the parallel, randomized trial of high initial dose versus low initial dose of snake antivenom for the treatment of neurotoxic envenoming.

Between April 2011 and October 2012, we assessed 194 snakebite victims with signs of envenoming, of whom 157 were enrolled into the trial (patient flow shown in Fig 1). Of these, 78 and 79 were randomised to the low and high initial dose groups, respectively. Two participants did not receive antivenom because one died before antivenom could be started, and the other had been wrongly randomised (absence of neurotoxicity). One patient in the high dose group withdrew consent. Finally, 154 patients could be included in the mITT analysis while 137 patients were included in the PP analysis.

The two treatment groups were similar with respect to baseline characteristics and sex ratio (Table 1). Thirty-two patients out of 154 (20·8%) were aged < 15 years. The severity of envenoming on admission was similar in both groups. The snake species was identified in 53 (34·4%) of 154 trial participants: 29 had been bitten by spectacled cobras (*Naja naja*), 20 by common kraits (*Bungarus caeruleus*), two by other kraits (*B*. *lividus* and *B*. *niger*), and two by other cobras (*Naja kaouthia* and *Naja* sp.). The distribution of snake species was balanced between treatment groups. Kraits caused more bites in Bharatpur than Damak (see S1 Table).

**Table 1:** Baseline demographic and epidemiological characteristics of trial participants. Figures are numbers of participants (percentage) unless stated otherwise.

Parameter		Overall N = 154	Low dose N = 76	High dose N = 78
Study Center	Damak	55 (35·7%)	27 (35·5%)	28 (35·9%)
	Charali	26 (16·9%)	12 (15·8%)	14 (18·0%)
	Bharatpur	73 (47·4%)	37 (48·7%)	36 (46·2%)
Sex	Female	80 (51·9%)	39 (51·3%)	41 (52·6%)
	Male	74 (48·1%)	37 (48·7%)	37 (47·4%)
Age (years)	Median (IQR)	28 (16–46)	26 (16–44)	32 (17–49)
Time to reach center (min)	Median (IQR)	75 (45–148)	66 (41–134)	80 (50–150)
	*Missing*	*7*	*2*	*5*
Neurotoxic score on admission	Mean ± sd	2·14 ± 1·18	2·21 ± 1·33	2·08 ± 1·01
Snake species	Unidentified	101 (65·6%)		
	Identified	53 (34·4%)		
	Cobra	31 (58%)	15 (58%)	16 (59%)
	Krait	22 (42%)	11 (42%)	11 (41%)

**Fig 2:**
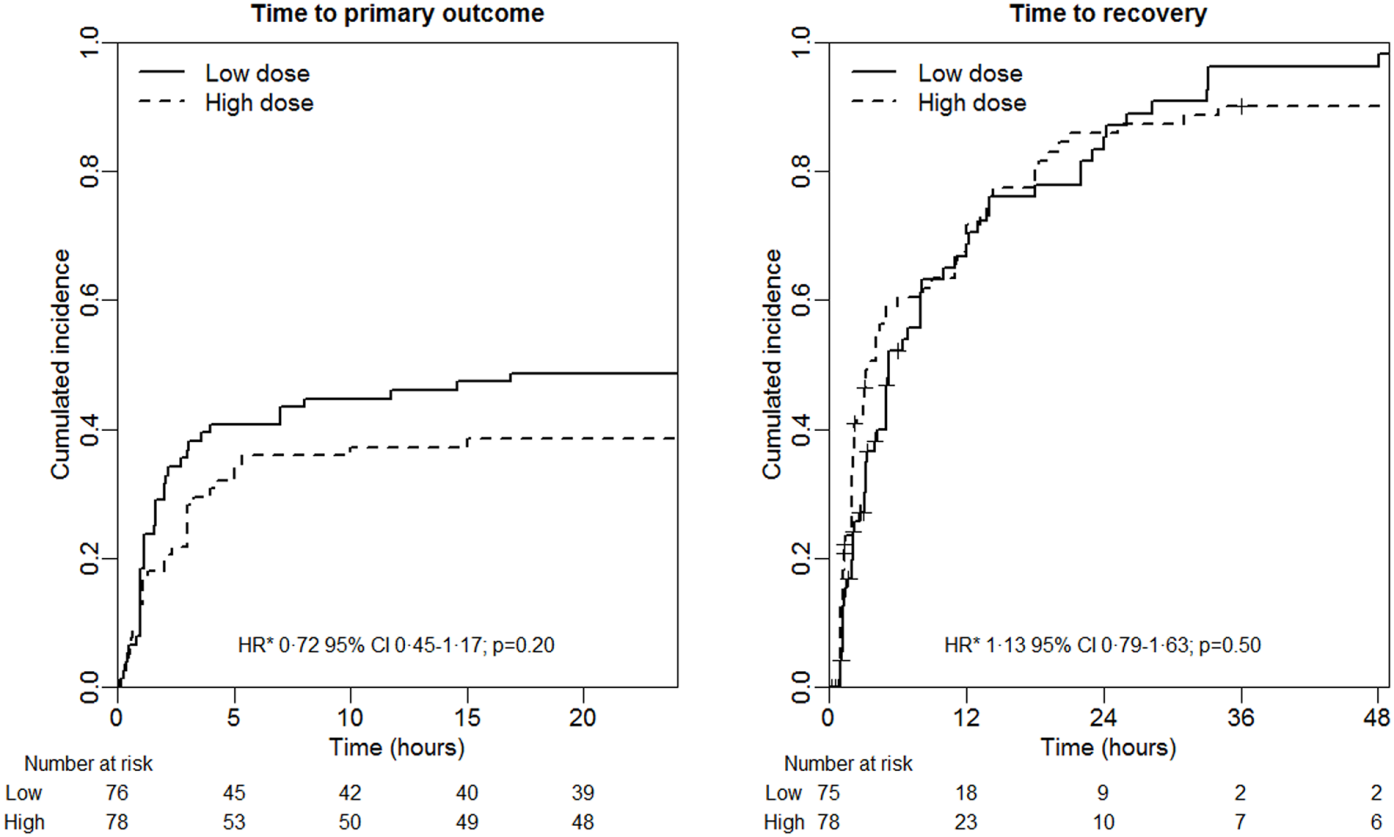
Cumulative incidence by study arm for primary outcome^1^ (left panel) and recovery^2^ (right panel) obtained with Kaplan-Meier survival estimator in 154 patients (modified intent-to-treat population).

Of the 154 participants included in the mITT analysis, 67 (43·5%) participants met the primary composite outcome definition of death, need for ventilation or worsening of neurotoxicity (Table 2). The proportion was slightly higher in the low vs. high initial dose group but this difference was not statistically significant (risk difference = 10·2%, 95%CI [-6·7; 27·1], p = 0·264). The proportions of patients who either died, required assisted ventilation or experienced worsening neurotoxicity did not differ among treatment groups. In a similar way, neither the time to primary outcome (HR = 0·72 95%CI [0·45; 1·17], p = 0·20) nor the time to recovery (HR = 1·13 95%CI [0·79; 1·63], p = 0·50) was significantly different between the two groups (Fig 2A and 2B). Similar results were obtained in the PP analysis (HR = 0·62 95%CI [0·37; 1·05], p = 0·07 and HR = 1·38 95%CI [0·94; 2·02], p = 0·17, respectively) and in a per centrer analysis (see S2 Table and S3 Fig).

**Table 2:** Effectiveness endpoints in modified intention-to-treat population. Figures are numbers of participants (percentage) unless stated otherwise.

	Low dose	High dose	Risk difference [95%CI]	p-value	HR* [95%CI]	p-value**
	n = 76	n = 78				
Primary composite outcome	37 (48·7%)	30 (38·5%)	10·2% [-6·7; 27·1]	0·264	0·72[0·45; 1·17]	0·199
Worsening toxicity^1^	31 (43·7%)	27 (35·5%)	8·1% [-9·0; 25·3]	0·401		
Need for ventilation	15 (19·7%)	13 (16·7%)	3·1% [-10·4; 16·6]	0·776		
Death	2 (2·6%)	7 (9·0%)	-6·3% [-14·9; 2·2]	0·167		
Number of vials, mean ± sd	11·0 ± 7·9	12·5 ± 3·9	1·5 [-0·5; 3·5]***	0·142		
< 10 vials	41 (53·9%)	0 (0%)				
10 to 15 vials	17 (22·4%)	70 (89·7%)				
> 15 vials	18 (23·7%)	8 (10·3%)				

^1^Seven patients had missing data for neurotoxicity score, 5 in the low dose group and 2 in the high dose group. All of these patients required ventilation so that a positive response to the primary composite outcome could be defined. It follows that no missing data remained for the primary composite outcome.

*adjusted for center;

**log-rank test stratified for center;

***mean difference [95%CI], p value from a Welch t test

The observed average number of vials consumed was higher in the high initial dose group (mean = 12·5) than in the low initial dose group (mean = 11·0), but the mean difference was not statistically significant (mean difference = 1·5 95%CI [-0·5; 3·5], p = 0·14). However, the percentage of patients having 16 or more vials was higher in the low initial dose group (24% vs. 10%, p = 0·0446) (Table 2).

We investigated the impact of the biting species on the effectiveness outcomes in the 53 patients for whom the species could be identified (Table 3). Patients bitten by kraits met the primary outcome more frequently, received more vials, recovered less often and when they did, the time for recovery was longer. The small number of victims for which the snake species could be identified precluded an effectiveness analysis by treatment group.

**Table 3:** Effectiveness endpoints by biting species.

Snake species	Cobras N = 31	Kraits (3 species) N = 22	Difference [95%CI]	p-value
Primary composite outcome, N (%)	8 (26%)	15 (68%)		0·004
Patients reaching full neurotoxic recovery, N (%)	29 (94%)	9 (41%)		<0·001
Time (h) to recovery, mean ± sd	5·0 ± 6·0	18·3 ± 12·0		0·0102
Number of vials, mean ± sd	8·9 ± 3·3	18·3 ± 6·8	9·4 [6·2; 12·6]	<0·0001

We also investigated the impact of the study centre on the effectiveness outcomes and found that the time to primary outcome was significantly shorter in Bharatpur than Damak, after adjustment for treatment arm (HR = 2·00 95%CI [1·2; 3·6], p = 0·014). The average number of vials consumed per patient was also higher in Bharatpur compared to the other two centers (mean difference = 3·7, 95%CI [1·8; 5·6], p = 0·0003).

A total of 137 patients (89%) reported ≥ 1 AE (Table 4) with no significant difference between high 73/78 (94%) and low dose 64/76 (84%) groups (risk difference = 9·4% 95%CI [-1·8; 20·5], p = 0·075). In 82 patients (53·2%) the AE was deemed related to antivenom treatment with no difference between high 42/78 (54%) vs. low dose 40/76 (53%) groups (p = 1). Seven out of 154 patients (5%) reported symptoms consistent with serum sickness (i.e., arthralgia occurring more than 7 days after antivenom treatment). In the PP analysis the proportion of patients experiencing at least one AE was higher in the high dose group (risk difference = 12·9% 95%CI [-0·6; 25·2], p = 0·0373). This difference in proportion was not specific to one type of AE. Among the 154 patients included in the mITT analysis, 18 experienced at least one SAE (Table 5), seven (9%) in the low dose group and 11 (14%) in the high dose group (p = 0·45). Among a total of 19 SAEs, seven were deemed definitely or probably related to the antivenom, two in the low dose group and five in the high dose group.

**Table 4:** Safety endpoints. Figures are numbers of participants (percentage) unless stated otherwise.

	All (n = 154)	Low dose (n = 76)	High dose (n = 78)	p-value
Patients reporting Adverse Events	137 (89%)	64 (84%)	73 (94%)	0·075
Patients reporting Serious Adverse Events	18 (12%)	7 (9%)	11 (14%)	0·45
Type of events reported				
*Skin and subcutaneous tissue disorders*	101 (65·6%)	49 (64·5%)	52 (66·7%)	0·866
Infected bite	62 (61·4%)	34 (44·7%)	28 (35·9%)	0·324
Pruritus, rash or angioedema	59 (58·4%)	29 (38·2%)	30 (38·5%)	1
*General disorders*	75 (48·7%)	35 (46·1%)	40 (51·3%)	0·524
Fever and chills	73 (47·4%)	35 (46·1%)	38 (48·7%)	0·750
*Gastrointestinal disorders*	51 (33·1%)	28 (36·8%)	23 (29·5%)	0·393
Epigastric discomfort	28 (18·2%)	16 (21·1%)	12 (15·4%)	0·408
Vomiting	23 (14·9%)	11 (14·5%)	12 (15·4%)	1
Abdominal pain	6 (3·9%)	4 (5·3%)	2 (2·6%)	0·439
*Respiratory*, *thoracic and mediastinal disorders*	39 (25·3%)	18 (23·7%)	21 (26·9%)	0·712
Tachypnoea	24 (15·6%)	10 (13·2%)	14 (17·9%)	0·507
Wheezing/laryngeal edema	13 (8·4%)	7 (9·2%)	6 (7·7%)	0·779
Respiratory failure	6 (3·9%)	0 (0%)	6 (7·7%)	0·028
*Nervous system disorders*	22 (14·3%)	8 (10·5%)	14 (17·9%)	0·250
Paraesthesia	11 (7·1%)	6 (7·9%)	5 (6·4%)	0·764
Headache	7 (4·5%)	1 (1·3%)	6 (7·7%)	0·117
*Musculoskeletal and connective tissue disorders*	22 (14·3%)	9 (11·8%)	13 (16·7%)	0·491
Myalgia	9 (5·8%)	3 (3·9%)	4 (5·1%)	1
Arthralgia^1^	7 (4·5%)	3 (3·9%)	4 (5·1%)	1

^1^ Late arthralgia: defined as occurring later than 7 days after treatment initiation

**Table 5:** List of serious adverse events (SAE) occurring in snakebite victims with neurotoxic signs randomized to either a low or a high initial dose of antivenom.

	Nature of the SAE	Seriousness criteria	Relationship to treatment	Outcome
1	Anaphylactic reaction	Life-threatening	Definitely related	Resolved
2	Anaphylactic reaction	Results in death	Probably related	Death
3	Anaphylactic reaction	Life-threatening	Definitely related	Resolved
4	Delayed anaphylactic reaction	Results in death	Probably related	Death
5	Anaphylactic reaction	Life-threatening	Definitely related	Resolved
6	Anaphylactic reaction	Life-threatening	Definitely related	Resolved
7	Gangrene of bitten finger	Prolonged hospitalization and permanent disability	Unlikely to be related	Resolved with sequelae
8	Respiratory failure	Results in death	Not related	Death
9	Cardiac arrest	Life-threatening	Definitely related	Resolved
10	Tracheostomy^1^	Results in death	Unlikely to be related	Not resolved
11	Sudden death after discharge (unexplained)	Results in death	Unlikely to be related	Not resolved
12	Anaphylactoid reaction	Life-threatening	Probably related	Resolved
13	Overdose^2^	Overdose	NA	NA
14	Death (unexplained reason)	Results in death	Unlikely to be related	Death
15	Death (overwhelming envenoming)	Results in death	Unlikely to be related	Death
16	Post-anoxic myoclonus	Prolonged hospitalization	Unlikely to be related	Recovered
17	Anaphylactoid reaction	Results in death	Probably related	Death
18	Respiratory failure	Results in death	Unlikely to be related	Death
19	Death (overwhelming envenomation)	Results in death	Unlikely to be related	Death

^1^Death consecutive to tracheostomy occurred in the same patient as the cardiac arrest.

^2^As per trial protocol, overdose of antivenom were to be considered as SAE and reported on expedited basis to the sponsor.

The incidence of anaphylactic reactions did not differ significantly before and after the implementation of the low-dose adrenaline pre-treatment in April 2012 (pruritus, rash or angiodema: 30/85 (35·3%) vs. 29/69 (42·0%) p = 0·491, wheezing/laryngeal oedema 10/85 (11·8%) vs. 3/69 (4·3%) p = 0·175, anaphylaxis 0/85 (0%) vs. 1/69 (1·5%) p = 0·448, cardiovascular shock 4/85 (4·7%) vs. 0/69 (0%) p = 0·128).

## Discussion

This study failed to demonstrate that a high initial dose of antivenom was more effective than a low initial dose in treating neurotoxic envenoming among Nepali patients. None of the components of the composite primary end point were significantly different in the higher dose group, but the data suggested that the rate of progression was slower in this group. The occurrence of AEs appeared slightly higher in the high dose group (statistical significance was not achieved in mITT but in PP analyses), however this difference was not clinically relevant.

Our study took place in two dedicated snakebite clinics, one in a rural area and the other in a small town, and in a referral hospital of a larger town. Thus, our study mirrored the routine management of snakebite victims in Nepal and, probably, most of South Asia. This and the low number of losses-to-follow-up increased our study’s external validity. Another significant strength was the ability to ascertain the biting species in a third of patients. Identifying the snake species is extremely challenging in South Asia because of the lack of robust methods [23], while patients’ descriptions are unreliable. The response to antivenom is highly dependent on the toxins of the biting snake. Thus, determining the biting species is key to giving the correct antivenom and anticipating the clinical course and potential complications. Other studies have achieved higher rates of species identification [12,13], thanks to ELISA-based methods against circulating venom components.

To our knowledge, this is the first robust RCT to compare different dose regimens of antivenom in the treatment of neurotoxic envenoming. Most published studies lacked a proper power calculation [7,10,34], were un-blinded [7,9,10] and/or used inappropriate or incomplete randomization [10,12,34]. Moreover, several studies mixed neurotoxic and haematotoxic envenoming or included patients with nonspecific manifestations like confusion or bradycardia [7–9]. A systematic review in 2015 deemed these studies to be of very low quality evidence [17]. There is currently no validated and internationally recommended protocol to monitor the clinical progression of neurotoxic envenoming. We developed a scoring system, based on objective, readily-assessable, clinical signs that may be used by staff in small health posts or clinics. Although it has yet to be formally validated, the intra- and inter- observer reliability of the scoring method was tested during the planning phase of the trial and found to be high. The calculated scores also showed consistency across different observers and over different time periods in retrospective analysis, adding confidence to our endpoint measurements.

Almost half of the trial participants either died, developed respiratory paralysis, or experienced a worsening of neurotoxicity despite the administration of antivenom. Antivenom effectiveness depends on its ability to neutralise the venom of the local snakes. Several medically-important snake species in Nepal are not covered covered by the Indian antivenom, while *E*. *carinatus* is not present in the country [23]. Although most species responsible for envenoming bites in the present study were the same as those whose venom is used to raise Indian antivenom, venom composition is known to vary within a species from region to region. The pre-clinical efficacy of Indian antivenoms against the venoms of Nepali neurotoxic species is unknown. Both the greater species diversity and geographical variation in venom composition could have contributed to the overall poor performance of the antivenom. Moreover, the utility of antivenom in the management of krait envenoming has long been questioned [35,36]. The most lethal neurotoxins of krait venoms, β-bungarotoxins, are presynaptic in their mode of action, irreversibly destroying motor nerve terminals. Thus, clinical recovery is slow because it depends on the regeneration of the neuromuscular junction [36]. Results of our subgroup analyses confirmed that, compared to the cobra victims, patients bitten by kraits deteriorated more frequently, recovered more slowly and required more vials of antivenom. These findings are consistent with the observation that, in Bharatpur hospital where krait bite envenoming predominates [23], patients had a worse prognosis than at the other study sites. We support calls for the establishment of regional venom banks of geographically representative snake populations [37] for the development of new, better-targeted antivenoms. It is also an essential pre-requisite of national regulatory authorities to test independently the effectiveness of marketed antivenoms, in line with WHO recommendations [5].

The absence of a significant difference between the high and the low-dose groups in the response to antivenom should not be interpreted as evidence of no benefit of a high initial dose. This absence of statistical significance is potentially due to a lack of power owing to a lower-than-targeted sample size, an optimistic hypothesized difference in the sample size calculation (the observed difference was only ~10% whereas we powered the study to detect a 20% difference), and the substantial proportion of krait bite envenoming in our study population. Convincing evidence of the benefit or superiority of the higher initial dose regimen would require a large study with a mortality endpoint and higher proportion of identified snakes, a very unlikely scenario for such a neglected disease. In view of the complexity of the dosing regimen recommended in the Nepal national guidelines, and since a high initial dose regimen does not result in a higher consumption of antivenom, the dose regimen recommended by WHO guidelines seems a reasonable preference for treating neurotoxic envenoming in Nepal and the rest of South Asia. Clinicians will have to balance the simplicity of administration of this regimen with a slight increase in the occurrence of adverse events.

## Supporting information

S1 Fig Neurotoxicity score used in the trial. Each sign scored one.
(DOCX)

S2 Fig Schematic diagram of antivenom dosing schedule used in the trial. (PDF)

S3 Fig Cumulative incidence by study arm and by centre for primary outcome obtained with Kaplan-Meier survival estimator in 137 patients (per protocol population). (DOCX)

S1 Table Distribution of snake species responsible for bites among study centers. (DOCX)

S2 Table Efficacy endpoints in modified per protocol population. Figures are numbers of participants (percentage) unless stated otherwise.
(DOCX)
