# Supplementary material for: Dose of antivenom for the treatment of snakebite with neurotoxic envenoming: Evidence from a randomised controlled trial in Nepal
Source: PLoS Negl Trop Dis. 2017 May 16;11(5):e0005612. doi: 10.1371/journal.pntd.0005612 (PMC5446183; doi:10.1371/journal.pntd.0005612)
Supplement: S1 Fig — Each sign scored one. (DOCX) [file pntd.0005612.s001.docx]

**S1 Figure: Neurotoxicity score used in the trial. Each sign scored one.**

| Sign | Present =1  Absent =0 |
| --- | --- |
| Inability to frown |  |
| Inability to retract upper eyelids on looking up (bilateral ptosis) |  |
| Inability to open the mouth |  |
| Inability to protrude the tongue beyond incisors |  |
| Inability to swallow |  |
| Broken neck sign |  |
| Skeletal muscle weakness |  |
| Gag reflex loss^1^ |  |
| Paradoxical breathing^1^ |  |
| **Total** |  |

^1^ severe signs
