# Supplementary material for: Dose of antivenom for the treatment of snakebite with neurotoxic envenoming: Evidence from a randomised controlled trial in Nepal
Source: PLoS Negl Trop Dis. 2017 May 16;11(5):e0005612. doi: 10.1371/journal.pntd.0005612 (PMC5446183; doi:10.1371/journal.pntd.0005612)

Low initial dose regimen

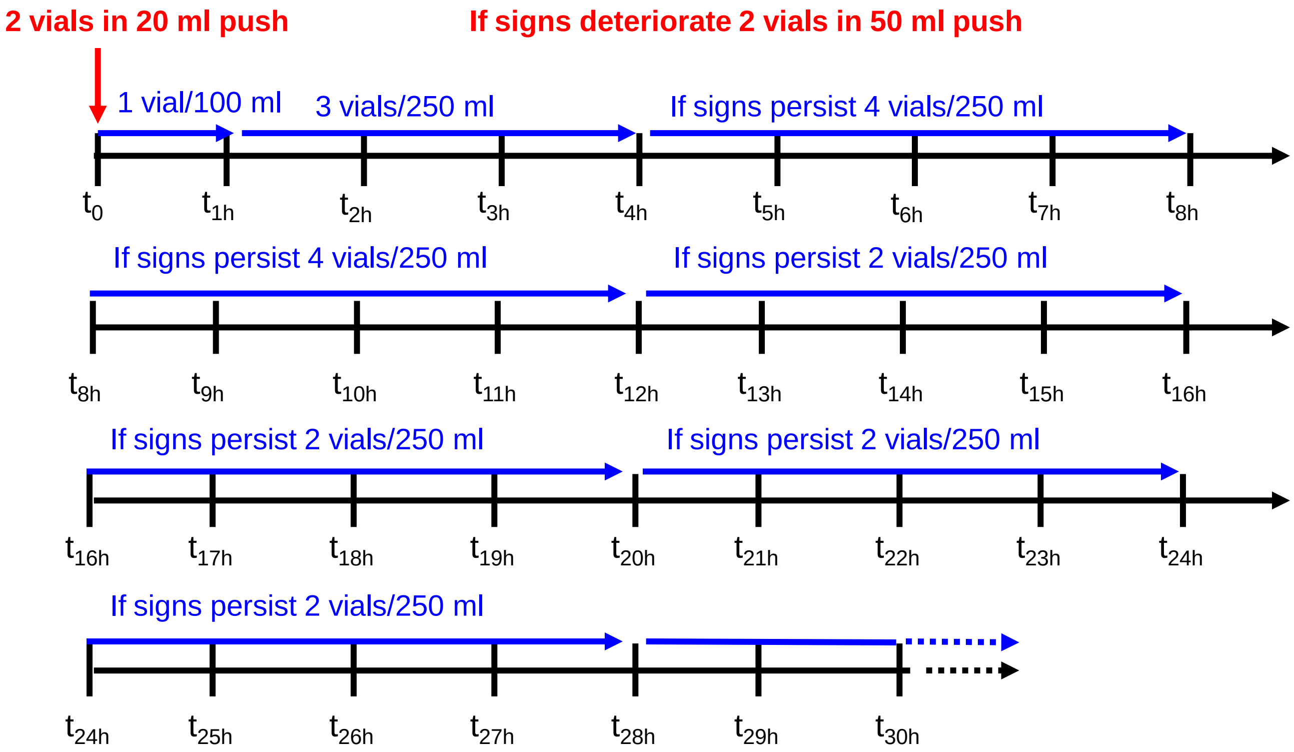

High initial dose regimen

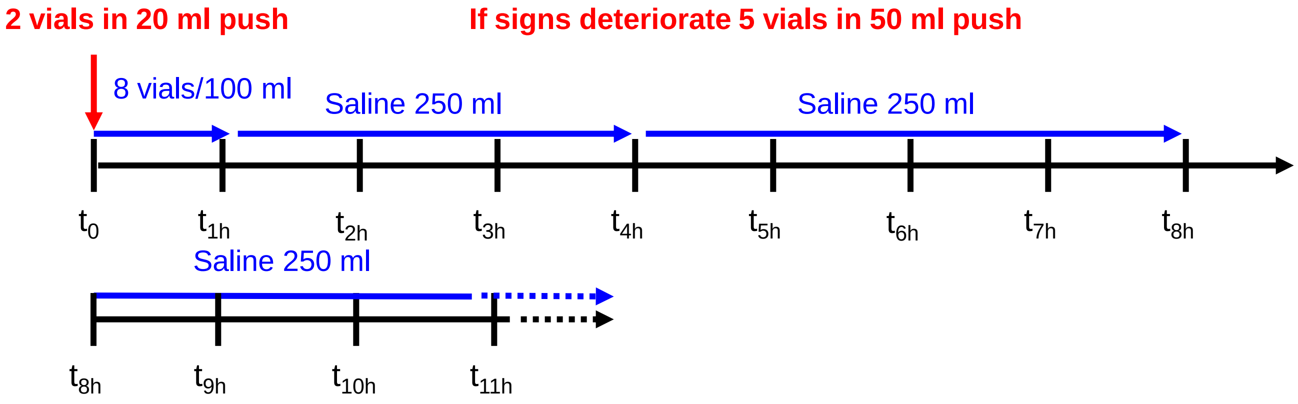

Supplement: S2 Fig — (PDF) [file pntd.0005612.s002.pdf]
