# Supplementary material for: Dose of antivenom for the treatment of snakebite with neurotoxic envenoming: Evidence from a randomised controlled trial in Nepal
Source: PLoS Negl Trop Dis. 2017 May 16;11(5):e0005612. doi: 10.1371/journal.pntd.0005612 (PMC5446183; doi:10.1371/journal.pntd.0005612)

**S3 Figure: Cumulative incidence by study arm and by centre for primary outcome obtained with Kaplan-Meier survival estimator in 137 patients (per protocol population)**


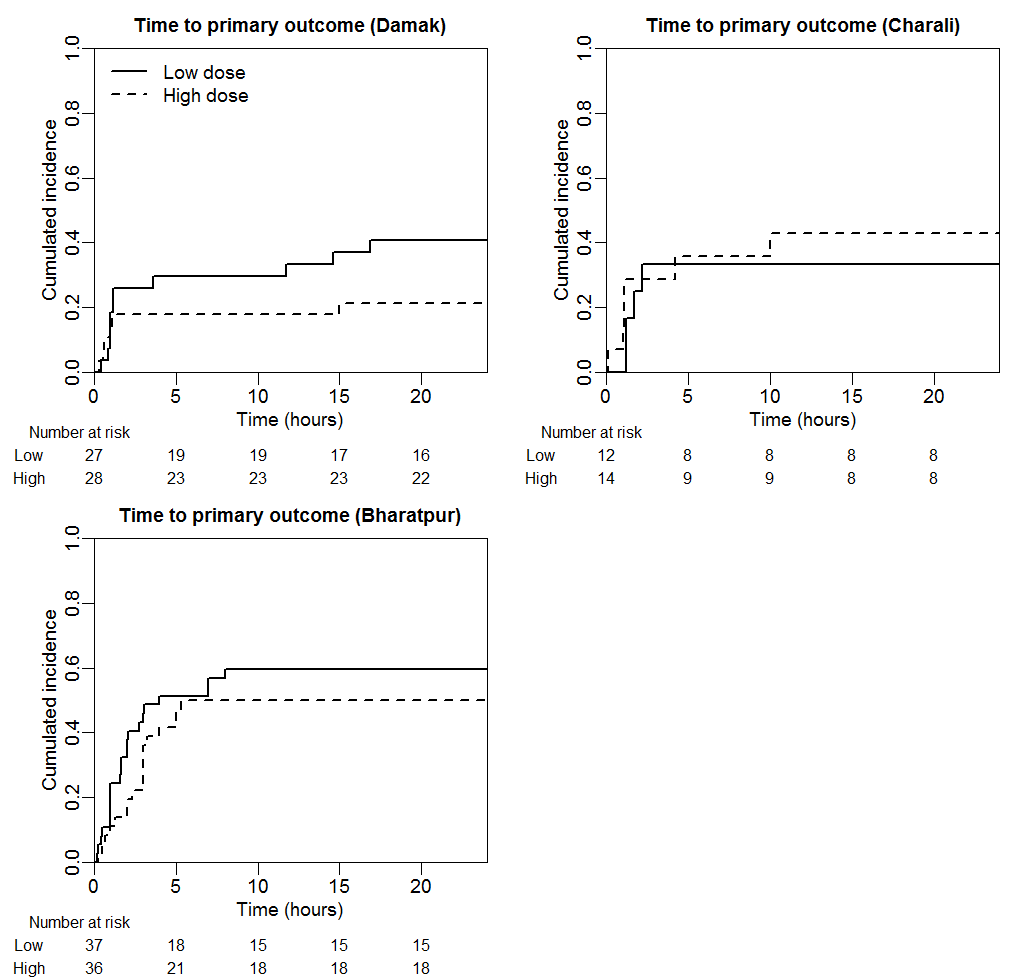

Supplement: S3 Fig — (DOCX) [file pntd.0005612.s003.docx]
