# Supplementary material for: Dose of antivenom for the treatment of snakebite with neurotoxic envenoming: Evidence from a randomised controlled trial in Nepal
Source: PLoS Negl Trop Dis. 2017 May 16;11(5):e0005612. doi: 10.1371/journal.pntd.0005612 (PMC5446183; doi:10.1371/journal.pntd.0005612)
Supplement: S1 Table — (DOCX) [file pntd.0005612.s004.docx]

**S1 Table: Distribution of snake species responsible for bites among study centres**

| Species | All  N=154 | Damak  N=55 | Charali  N=26 | Bharatpur  N=73 |
| --- | --- | --- | --- | --- |
| Cobra | 29 | 22 (100%) | 1 (33·3%) | 8 (28·6%) |
| Krait | 22 | 0 (0%) | 2 (66·7%) | 20 (71·4%) |
| missing | 103 | 33 | 23 | 45 |
