# Supplementary material for: Dose of antivenom for the treatment of snakebite with neurotoxic envenoming: Evidence from a randomised controlled trial in Nepal
Source: PLoS Negl Trop Dis. 2017 May 16;11(5):e0005612. doi: 10.1371/journal.pntd.0005612 (PMC5446183; doi:10.1371/journal.pntd.0005612)
Supplement: S2 Table — Figures are numbers of participants (percentage) unless stated otherwise. (DOCX) [file pntd.0005612.s005.docx]

**S2 Table: Efficacy endpoints in modified per protocol population.** Figures are numbers of participants (percentage) unless stated otherwise

|  | Low dose | High dose | Risk difference [95%CI] | p-value | HR* [95%CI] | p-value** |
| --- | --- | --- | --- | --- | --- | --- |
| *PP population* | n= 65 | n= 72 |  |  |  |  |
| Primary composite outcome | 31 (47·7%) | 25 (34·7%) | 13·0% [-4·9; 30·8] | 0·171 | 0·62 [0·37; 1·05] | 0·07 |
| Worsening toxicity^1^ | 25 (41·7%) | 22 (31·4%) | 10·2% [-7·9; 28·3] | 0·304 |  |  |
| Need for ventilation | 13 (20·0%) | 8 (11·1%) | 8·9% [-4·7; 22·5] | 0·228 |  |  |
| Death | 2 (3·1%) | 4 (5·6%) | -2·5% [-10·7; 5·7] | 0·683 |  |  |

PP: Per protocol
